# Supplementary material for: Chromothripsis during telomere crisis is independent of NHEJ, and consistent with a replicative origin
Source: Genome Res. 2019 May;29(5):737–49. doi: 10.1101/gr.240705.118 (PMC6499312; doi:10.1101/gr.240705.118)
Supplement: Supplemental Material [file supp_gr.240705.118_Supplemental_file_1.zip › contigs/annotated_contigs/DB50/contig.2.DB50_length_335_mean_cov_4.31641791045.docx]

**DB50_length_335_mean_cov_4.31641791045**

```
TGACTTCCATTTGTGAAAGTAGAAGA|CTAATATCATGGCTGTTTTCTATGCTTACAAGAAAGGAAACCCACAAAATGGAAGGATCCTT
 >chr10:8380222-8380248 + E=3e-04  >chr10:7336125-7336434 + E=2e-175
CCAATAAAAAGCACGCCAACTCACCCAGTGCATCTGTATGGGATAAAAGGATGAGACGTAAACCCTTGGTCCATGTACATCTCACCTTC

CTCCAGTGAAACCTCATCCTAAACCTGTAATATATGATGTTGTAGAATTTATCTCTGACCCTAGCATGTAACAGTAGGACTGGGCAGAA

CCCACCTTGCATTGTACAAGTATCTAGACTCTATCTGGACAGCCATGTGCCCAGCTACATCTATTTCCA
```
